# Virtual services in the health sciences library: a handbook

**DOI:** 10.29173/jchla29735

**Published:** 2023-12-01

**Authors:** Alison Manley

**Affiliations:** Health Sciences Librarian, Library Services Horizon Health Network, Miramichi, NB, Canada

Scull, AR. **Virtual services in the health sciences library: a handbook**. Lanham, MD: Rowman & Littlefield; 2022. Hardcover: 160 p. ISBN: 978-1-5542-4. Price: USD$135.00. Available from: https://rowman.com/ISBN/9781538155424/Virtual-Services-in-the-Health-Sciences-Library-A-Handbook

With the growing hunger in the profession for how to best implement virtual services in libraries and the reality that most libraries had to figure out how to do that on a moment’s notice in 2020, as a result of the COVID-19 pandemic, *Virtual services in the health sciences library: a handbook*, edited by Amanda R. Scull, is a welcome addition to the literature on virtual library services. This handbook covers the current moment in virtual services: programs implemented due to COVID-19 closures and programs that have been refined since the acute phase of the pandemic. The time is right for this handbook to make its way into any health sciences library worker’s hands. It spurs us to think about how we can adapt these ideas into our own contexts. The handbook deals with health sciences libraries of all kinds, from large academic medical centres to small rural hospital libraries. I appreciate this attention to the different types of health sciences library practice environments, as it can be challenging to find handbooks that deal with the differing sizes and types of health sciences libraries.

The contributors to *Virtual services* are all involved in library work at health sciences libraries, with varying experience. Some of the contributors have lengthy careers in health sciences libraries, while others are newer to the field, providing a well-rounded lens through which to explore virtual services. The editor and author of one chapter, Amanda Scull, is the head of education and information services at the Dartmouth College Biomedical Libraries, while other contributors run the gamut of directors, liaison librarians, communications specialists, and managers.

*Virtual services* is divided into two sections: Part I is titled Information services, engagement, and access, with four chapters. A second slightly more robust section is titled Research, instruction, and clinical support, with six chapters. However, there is a considerable amount of overlap in the programs described and evaluated in each section, and the categorization is not strictly necessary. Opening the book is a preface that sets the tone as a project birthed specifically from the pandemic, and while this may ultimately date the book in the near future, it is invaluable context. As Scull writes:

In some ways, the pandemic showed us ways in which virtual services could be even better than in-person services for providing prompt patron services. All this is to say that the pandemic has been the impetus, but it is not the endpoint.

The handbook covers both internal and external remote issues, such as creating a stronger online brand to meet library users online (Countway Library, Harvard), managing virtual staff development (Dartmouth College Biomedical Libraries), and bringing a rural hospital library system online (Hunter-Rice Health Sciences Library). The diversity of ways to manage virtual services and recognizing that we are providers of services to ourselves and our staff are happy addition to the handbook as well as being very useful. Each chapter is written practically, with a clear outline, recommendations, and grounding in previous literature. This handbook is a project that clearly had strong guidelines for the chapters. The result is a pleasantly cohesive set of studies.

I was incredibly pleased with *Virtual services*. So often I read handbooks like this and come away disappointed because they do not address different settings, or they bill themselves as more universal than they actually are. This is a handbook with both Canadian and American contributors, a diversity of health sciences library settings, and practically written chapters that cover a wide variety of topics. I see myself consulting it in the future as I look to modify or implement new virtual services in my library. This is a strong and welcome addition to the virtual services library literature.

